# Contribution of alpha- and beta-defensins to lung function decline and infection in smokers: an association study

**DOI:** 10.1186/1465-9921-7-76

**Published:** 2006-05-15

**Authors:** Alison M Wallace, Jian-Qing He, Kelly M Burkett, Jian Ruan, John E Connett, Nicholas R Anthonisen, Peter D Paré, Andrew J Sandford

**Affiliations:** 1 James Hogg iCAPTURE Centre for Cardiovascular and Pulmonary Research, University of British Columbia, Vancouver, Canada; 2 Division of Biostatistics, School of Public Health, University of Minnesota, Minneapolis, USA; 3 Faculty of Medicine, University of Manitoba, Winnipeg, Canada

## Abstract

**Background:**

Alpha-defensins, which are major constituents of neutrophil azurophilic granules, and beta-defensins, which are expressed in airway epithelial cells, could contribute to the pathogenesis of chronic obstructive pulmonary disease by amplifying cigarette smoke-induced and infection-induced inflammatory reactions leading to lung injury. In Japanese and Chinese populations, two different beta-defensin-1 polymorphisms have been associated with chronic obstructive pulmonary disease phenotypes. We conducted population-based association studies to test whether alpha-defensin and beta-defensin polymorphisms influenced smokers' susceptibility to lung function decline and susceptibility to lower respiratory infection in two groups of white participants in the Lung Health Study (275 = fast decline in lung function and 304 = no decline in lung function).

**Methods:**

Subjects were genotyped for the alpha-defensin-1/alpha-defensin-3 copy number polymorphism and four beta-defensin-1 polymorphisms (G-20A, C-44G, G-52A and Val38Ile).

**Results:**

There were no associations between individual polymorphisms or imputed haplotypes and rate of decline in lung function or susceptibility to infection.

**Conclusion:**

These findings suggest that, in a white population, the defensin polymorphisms tested may not be of importance in determining who develops abnormally rapid lung function decline or is susceptible to developing lower respiratory infections.

## Background

Chronic obstructive pulmonary disease (COPD) is characterized by irreversible airflow obstruction due to chronic inflammation. COPD is closely related to cigarette smoking, which is the main environmental risk factor. Longitudinal studies show that only a minority of cigarette smokers develop airflow limitation,[1] suggesting that other environmental or genetic factors are important. Family and twin studies provide further evidence that genetic factors play a key role in the etiology of this disease.[2,3] Inherited deficiency of alpha-1 antitrypsin is associated with COPD; other genetic risk factors remain to be identified.

Defensins are small (29–47 amino acids) cationic microbicidal peptides that form part of both the innate and adaptive immune responses. Defensins show activities against Gram-positive and Gram-negative bacteria, fungi, yeast, and enveloped viruses.[4] Defensins are known primarily for their antimicrobial activities; however, the scope of their activities extends beyond immune responses and some of these functions could contribute to lung injury. [5-9]

Based on a characteristic three-dimensional fold and a 6-cysteine/3-disulfide pattern, human defensins are divided into two groups, alpha-defensins and beta-defensins.[10] Three closely related alpha-defensins, alpha-defensin-1 (DEFA1), DEFA2, and DEFA3, are major constituents of neutrophil azurophilic granules (30–50% of the total protein content)[11] and play a role in antimicrobial activity and inflammation in the lung. Beta-defensin-1 (DEFB1) is constituatively expressed in airway epithelia and plays an important role in mucosal immunity in the lung.[12,13]

In humans, five alpha-defensins and at least four beta-defensins are clustered in a 450 kb region on chromosome 8p23.[14,15] DEFA1, DEFA2, and DEFA3 differ by only one amino acid. DEFA2 is the proteolytic product of DEFA1 and/or DEFA3; DEFA1 and DEFA3 are encoded by separate genes.[16] Due to a copy number polymorphism, individuals can inherit variable copy numbers of the DEFA1 and DEFA3 genes, and the DEFA3 gene is often absent.[16] Genetic variation in the DEFB1 gene is associated with COPD phenotypes in Japanese [17] and Chinese [18] populations.

We determined whether inheritance of both DEFA1 and DEFA3 genes, rather than the DEFA1 gene only, was associated with fast decline in lung function. In addition, we determined the frequency of four DEFB1 polymorphisms in the Lung Health Study participants. We also investigated the relationship between the defensin polymorphisms and smokers' susceptibility to lower respiratory infection.

## Methods

### Study subjects

Subjects were selected from participants in Phase I of the National Heart, Lung, and Blood Institute (NHLBI) Lung Health Study. Details of the study have been previously published.[19] Briefly, study participants were current smokers, 35–60 years of age, who had mild to moderate airflow obstruction (FEV1 55–90% predicted and FEV1/FVC ≤ 0.70). The primary outcome variable was rate of decline in post-bronchodilator FEV1 over a follow-up period of five years. Lower respiratory infection rate was quantified using the number of self-reported visits to a physician and days in bed for lower respiratory infection at each follow-up. Of the 3216 continuing smokers in this cohort, 275 were chosen with a fast decline in lung function (decline in percent predicted FEV1 > 3.0% per year), and 304 were selected with no decline in lung function over the same period (increase in percent predicted FEV1 > 0.4% per year). All 579 selected participants were white and non-Hispanic. In addition, 27 African American Lung Health Study participants were genotyped; the data were used to determine the prevalence of genotypes across racial groups.

### Genotyping

Restriction fragment length polymorphism analysis was performed in order to distinguish the DEFA1 and DEFA3 genes.[14] An amplicon of 950 bp was generated by 35 cycles of PCR using the sense primer 5'-CAGCGGACATCCCAGAAGTGG and the antisense primer 5'-GCGTTTTGGTACGTGTATCC. PCRs were performed in a total reaction volume of 20 μL with 100 ng of genomic DNA, 0.5 U *Taq *polymerase (Invitrogen), 10X PCR buffer (Invitrogen), 3 mM Mg^2+^, 0.4 μM forward and reverse primers, and 200 μM dNTPs. After the initial denaturation at 95°C for 15 min, the reaction mixture was subjected to 35 cycles of 94°C for 30 s, annealing for 30 s at 57°C, and 72°C for 30 s followed by the final extension at 72°C for 5 min. After PCR, 20 μL of the reaction mixture was digested with 1.25 U *Hae *III restriction endonuclease (New England BioLabs Inc., Beverly, MA). The digest mixture was resolved on a 2% agarose gel stained with ethidium bromide. DNA from individuals with DEFA1 only produced two bands, one at 300 bp and one at 650 bp, and individuals with both DEFA1 and DEFA3 produced all three bands. Genotyping of the DEFB1 polymorphisms at positions -20, -44, and -52 in the 5' untranslated region were performed by restriction fragment length polymorphism analysis.[14] An amplicon of 260 bp was generated by 35 cycles of PCR using the sense primer 5'-GTGGCACCTCCCTTCAGTTCCG and the antisense primer 5'-CAGCCCTGGGGATGGGAAACTC. PCRs were performed in a total reaction volume of 60 μL with 100 ng of genomic DNA, 0.5 U *Taq *polymerase (Invitrogen), 10X PCR buffer (Invitrogen), 1.5 mM Mg^2+^, 0.75 μM forward and reverse primers, and 200 μM dNTPs. After the initial denaturation at 95°C for 15 min, the reaction mixture was subjected to 35 cycles of 94°C for 30 s, annealing for 30 s at 67°C, and 72°C for 30 s followed by the final extension at 72°C for 5 min. After PCR, 20 μL of the reaction mixture was digested with 2 U *Scr *FI restriction endonuclease (New England BioLabs Inc.) to detect variation at position -20. The digest mixture was resolved on a 3% agarose gel stained with ethidium bromide. DNA from individuals with the homozygous G genotype (GG) produced three bands at 136 bp, 118 bp, and 6 bp; the homozygous A genotype (AA) produced two bands, one at 254 bp and one at 6 bp; and the heterozygous genotype (GA) produced all four bands. 20 μL of the reaction mixture was digested with 4 U *Hga *I restriction endonuclease (New England BioLabs Inc.) to detect variation at position -44. The digest mixture was resolved on a 3% agarose gel stained with ethidium bromide. DNA from individuals with the homozygous C genotype (CC) produced two bands, one at 71 bp and one at 189 bp; the homozygous G genotype (GG) produced three bands at 71 bp, 30 bp, and 159 bp; and the heterozygous genotype (CG) produced all three bands. 20 μL of the reaction mixture was digested with 2 U *Nla *IV restriction endonuclease (New England BioLabs Inc.) to detect variation at position -52. The digest mixture was resolved on a 3% agarose gel stained with ethidium bromide. DNA from individuals with the homozygous G genotype (GG) produced three bands at 108 bp, 122 bp, and 30 bp; the homozygous A genotype (AA) produced two bands, one at 230 bp and one at 30 bp; and the heterozygous genotype (GA) produced all four bands. The DEFB1 Val38Ile polymorphism was analyzed as previously described.[17] Template-free controls and known genotype controls were included in each experiment. Twenty samples were selected at random and sequenced to confirm the genotyping protocols. Genotypes were assigned by two independent investigators who were unaware of the patients' identities and phenotypes. Inconsistencies were resolved by two additional genotyping reactions.

### Statistical analysis

Hardy-Weinberg equilibrium tests and linkage disequilibrium estimation were performed using Arlequin version 2.0.[20] Haplotype frequencies were estimated using PHASE version 2.0.[21,22] The chi-square test was used to test for association of genotype with decline in lung function status. Logistic regression was then performed to adjust for the potential confounding factors of age, sex, smoking history (pack-years), methacholine responsiveness, and initial level of lung function (pre-bronchodilator FEV1 percent predicted). Both analyses were performed using JMP Version 5.1. The infection outcomes of physician visits/year and days in bed/year are highly right-skewed, with a high proportion of subjects having no infection outcomes. To handle this type of data, a two-part model was used.[23,24] The first part deals with the spike of observations at zero and models which factors contribute to a person developing infection-related outcomes. The second part models the severity, duration (days in bed/year), or frequency (physician visits/year) of the infection related outcomes in those who had an outcome. For the first part of the model, logistic regression was used to test for association with the presence of at least one infectious event in a year. The data for the second part consists only of those with an infection related outcome and is highly skewed. Generalized Linear Model regression, assuming gamma distributed observations and a log link,[25] was then used to test for association of genotype with the average number of infectious events per year in those having any such events. For both regression analyses, rate of decline was included as a factor and both were performed using R . All haplotype analyses were performed using a contributed R program called Hapassoc, which tests for haplotype-phenotype association when haplotype phase is unknown.[26,27] The power calculator used is available through the UCLA Department of Statistics . Power calculations for a sample size of n = 275 cases and n = 304 controls were performed using a two sided test with the observed allele frequencies, alpha = 0.05, and beta = 0.80. Statistical significance was defined at the 5% level.

## Results

### General characteristics

The study population consisted of 579 white and non-Hispanic smokers; 275 smokers with a fast decline in lung function and 304 smokers with no decline in lung function over the five year study period. The characteristics of the study group are described in Table 1. Age, sex, smoking history (pack-years), baseline FEV1, and methacholine response differed significantly between the two groups.

**Table 1:** Description of the study population.

**Variable**	**Fast decline (n = 275)**	**No decline (n = 304)**	p Value*
Age, yr†	49.5 (6.4)	47.6 (6.9)	0.0007
Sex, n (%)	163 men (59)	203 men (67)	0.06
Smoking history, pk yrs†‡	43.3 (19.1)	38.3 (18.1)	0.0005
Baseline FEV1, % predicted†§	72.7 (8.9)	75.7 (8.1)	<0.0001
Methacholine response†ll	-23.4 (32.7)	-7.5 (14.0)	<0.0001

* p Values derived from Wilcoxon test or chi-square analysis.

† Mean (SD).

‡ Number of packs of cigarettes smoked per day × number of years of smoking.

§ Lung function at the start of the study as measured percent predicted FEV1 (postbronchodilator).

ll Measurement of the responsiveness of the airways to methacholine expressed as percent decline in FEV1 per final cumulative dose of methacholine administered[36] Methacholine response is strongly associated with rate of decline in lung function[37,38]

### DEFA1 and DEFA3 genes and rate of decline in lung function

The number of individuals who had a copy of the DEFA1 gene only (n = 64; 31 fast decline and 33 no decline), rather than copies of both DEFA1 and DEFA3 genes (n = 515; 244 fast decline and 271 no decline), was not significantly different in the fast decline in lung function group (11.8%) compared with the no decline in lung function group (10.9%), p = 0.87. No individuals in the study group inherited a copy of the DEFA3 gene alone. Logistic regression modeling to adjust for confounding variables confirmed the lack of association (p = 0.99).

### DEFB1 gene polymorphisms and rate of decline in lung function

Genotypic frequencies of the three DEFB1 SNPs (G-20A, C-44G, and G-52A) analyzed are given in Table 2. The observed distribution of all three genotypes was consistent with Hardy-Weinberg equilibrium. Univariate analysis did not suggest any significant associations with the genotypes and rate of decline in lung function. Logistic regression modeling to adjust for confounding variables confirmed the lack of association (G-20A, p = 0.82; C-44G, p = 0.66; and G-52A, p = 0.76). Subjects were also genotyped for the DEFB1 Val38Ile SNP. Only one heterozygous subject was detected, suggesting that this polymorphism is very rare (<1%), at least in whites.

**Table 2:** Beta-defensin-1 genotypic frequencies by fast decline or no decline in lung function status. Frequencies shown, n (%).

**Polymorphism**	**Phenotype**	**Genotype**		
G-20A		GG	GA	AA
(rs11362)	Fast decline	83 (30.2)	131 (47.6)	61 (22.2)
	No decline	91 (29.9)	150 (49.3)	63 (20.7)
			*p = 0.89	

C-44G		CC	CG	GG
(rs1800972)	Fast decline	177 (64.4)	85 (30.9)	13 (4.7)
	No decline	198 (65.1)	91 (29.9)	15 (4.9)
			*p = 0.97	

G-52A		GG	GA	AA
(rs1799946)	Fast decline	107 (38.9)	128 (46.6)	40 (14.6)
	No decline	130 (42.8)	128 (42.1)	46 (15.1)
			*p = 0.55	

* p Values derived from chi-square test.

### DEFB1 linkage disequilibrium and haplotype association analysis

There was strong linkage disequilibrium between the three DEFB1 SNPs in the 5' untranslated region (Table 3). Seven haplotypes were revealed. The estimated haplotype frequencies in the two Lung Health Study groups are given in Table 4. Univariate analysis did not suggest any significant association between haplotypes and rate of decline in lung function. The three most common haplotypes (GCA, ACG, and GGG) were detected in two other studies and were the only haplotypes reported by those studies.[17,28]

**Table 3:** Beta-defensin-1 linkage disequilibrium.

	**D' (D/Dmax)**	**r^2^**	p Value*
G-20A and G-52A	0.75	0.29	<0.00001
G-20A and C-44G	0.88	0.17	<0.00001
C-44G and G-52A	0.93	0.13	<0.00001

* p Values derived from chi-square test.

**Table 4:** Beta-defensin-1 haplotypes and association with rate of decline in lung function.

**Imputed haplotypes (-20/-44/-52)**	**Fast decline, n (%)**	**No decline, n (%)**	p Value*
ACA	24 (4.4)	18 (3.0)	0.26
GCA	181 (32.9)	201 (33.1)	
GGA	3 (1.0)	1 (<1.0)	
ACG	223 (40.6)	257 (42.3)	
GCG	11 (2.0)	11 (1.8)	
AGG	6 (1.1)	1 (<1.0)	
GGG	102 (18.6)	119 (19.6)	

* p Value for global haplotype association calculated with a Wald statistic.

### Secondary outcomes analysis

Statistical analysis did not suggest any significant associations between genotypes (Table 5) or haplotypes (Table 6) and infection outcomes.

**Table 5:** Defensin genotypes and infection outcomes.

**Polymorphism**	**Genotype**	**Physician visits‡§**	Part 1 p Value*	**Part 2 p Value†**	**Days in bed‡ll**	Part 1 p Value*	**Part 2 p Value†**
DEFA1/DEFA3							
	DEFA1 only	0.22 (0.07)	0.23	0.05	0.38 (0.16)	0.60	0.36
	DEFA1/DEFA3	0.28 (0.02)			0.51 (0.06)		

DEFB1 G-20A							
	GG	0.27 (0.04)	0.85	0.88	0.49 (0.10)	0.86	0.98
	GA	0.26 (0.03)			0.48 (0.08)		
	AA	0.30 (0.05)			0.54 (0.11)		

DEFB1 C-44G							
	CC	0.28 (0.03)	0.81	0.42	0.53 (0.07)	0.93	0.14
	CG	0.27 (0.04)			0.44 (0.10)		
	GG	0.18 (0.10)			0.28 (0.24)		

DEFB1 G-52A							
	GG	0.29 (0.04)	0.67	0.62	0.47 (0.08)	0.81	0.29
	GA	0.25 (0.03)			0.47 (0.08)		
	AA	0.30 (0.06)			0.62 (0.14)		

Definition of abbreviations: DEFA = alpha-defensin; DEFB = beta-defensin.

*Part 1 of the two-part model involves modeling whether an individual has any physician visits or any days in bed due to lower respiratory infection. P values for global haplotype association derived from logistic regression analysis. Decline in lung function status was included as a factor in the analysis.

†Part 2 of the two-part model involves modeling the average number of physician visits or days in bed due to lower respiratory infection given that it is greater than zero. P values for global haplotype association derived from gamma regression analysis. Decline in lung function status was included as a factor in the analysis.

‡Mean (SE).

§Mean number of visits to a physician for lower respiratory infections per year.

llMean number of days kept in bed for lower respiratory infections per year.

**Table 6:** Beta-defensin-1 haplotypes and infection outcomes.

**Haplotype**	**Physician visits‡§**	Part 1 p Value*	**Part 2 p Value†**	**Days in bed‡ll**	Part 1 p Value*	**Part 2 p Value†**
ACA	0.26 (0.08)	0.84	0.88	0.60 (0.19)	0.40	0.10
GCA	0.27 (0.03)			0.50 (0.06)		
GGA	0.50 (0.27)			2.50 (0.63)		
ACG	0.28 (0.02)			0.50 (0.06)		
GCG	0.45 (0.11)			1.00 (0.27)		
AGG	0.23 (0.20)			0.60 (0.48)		
GGG	0.24 (0.04)			0.36 (0.08)		

*Part 1 of the two-part model involves modeling whether an individual has any physician visits or any days in bed due to lower respiratory infection. P values derived from the Wald statistic based on logistic regression results from Hapassoc. Decline in lung function status was included as a factor in the analysis.

†Part 2 of the two-part model involves modeling the average number of physician visits or days in bed due to lower respiratory infection given that it is greater than zero. P values derived from the Wald statistic based on gamma regression results from Hapassoc. Decline in lung function status was included as a factor in the analysis.

‡Mean (SE).

§Mean number of visits to a physician for lower respiratory infections per year.

llMean number of days kept in bed for lower respiratory infections per year.

### Alpha-defensin and beta-defensin polymorphisms in different racial groups

We determined the prevalence of genotypes across white and African American participants of the Lung Health Study and found statistically significant differences between racial groups (Table 7).

**Table 7:** Frequency of defensin polymorphisms by racial group.

**Polymorphism**	**Genotype**	**White (n = 579)†**	**African American (n = 27)†**	p Value*
DEFA1/DEFA3				
	DEFA1 only	11.1	22.2	0.08
	DEFA1/DEFA3	88.9	77.8	

DEFB1 G-20A				
	GG	30.1	44.4	0.21
	GA	48.5	44.5	
	AA	21.4	11.1	

DEFB1 C-44G				
	CC	64.8	100.0	0.0008
	CG	30.4	0.0	
	GG	4.8	0.0	

DEFB1 G-52A				
	GG	40.9	22.2	0.001
	GA	44.2	37.1	
	AA	14.9	40.7	

Definition of abbreviations: DEFA = alpha-defensin; DEFB = beta-defensin.

* p Value derived from chi-square test.

†Overall observed SNP frequency (%).

## Discussion

We investigated the role of alpha-defensin and beta-defensin polymorphisms in promoting FEV1 decline in smokers with COPD and the influence on smokers' susceptibility to infection. Mars and coworkers[16] have shown that individuals can inherit variable copy numbers of the DEFA1 and DEFA3 genes, and that the DEFA3 gene is often absent. Several polymorphisms of beta-defensin-1 have been reported. [28-31] The frequency of polymorphisms in the coding region is low compared with polymorphisms in the promoter and untranslated regions. Of the reported polymorphisms, three polymorphisms in the 5' untranslated region at positions -20, -44, and -52 of the DEFB1 gene have minor allele frequencies greater than twenty percent in a white population.[28] The SNP at position -20 (G-20A) results in the formation of an nuclear factor-kappaB transcription factor-binding sequence; however, as DEFB1 is constituatively expressed, the functional impact is unclear.[30] It has been demonstrated that DEFB1 function is compromised in cystic fibrosis.[32] The SNP at position -44 (C-44G) is associated with *Candida *carriage in type I diabetics.[33] Variation in the DEFB1 gene is associated with asthma,[31] a condition that shares some underlying characteristics with COPD. The frequency of a SNP in exon 2 (Val38Ile) of the DEFB1 gene is significantly greater in male Japanese patients with COPD compared with healthy controls.[17] Our data indicate that this polymorphism is very rare in whites. More recently, a C-44G polymorphism, but not the Val38Ile polymorphism, was associated with COPD in a Chinese population.[18]

Defensins could play a role in the pathogenesis of COPD and are excellent candidate genes for COPD association studies due to their expression patterns, location, and function. First, DEFA1–3 are expressed in neutrophils and DEFB1 is expressed in airway epithelial cells. Second, members of the alpha-defensin and beta-defensin families are located on chromosome 8p23, a genomic region linked to the development of airflow obstruction in smokers.[34] Finally, defensins may contribute to the pathogenesis of COPD by amplifying the cigarette smoke-induced and the infection-induced inflammatory reactions, which may lead to lung tissue injury.

We conducted a population-based association study to determine whether alpha-defensin and beta-defensin polymorphisms influence smokers' susceptibility to lung function decline in a large population of white individuals. The present genetic association study fulfills the major criteria for robust association studies[35] and has the following strengths: 1) defensins are excellent COPD candidate genes because defensins mediate inflammation and response to infection in the lung; 2) we included only white subjects in the association study in order to minimize the potential effects of population stratification; 3) our study is a large association study involving 579 individuals; 4) linkage data from a previous study suggests that 8p23 contains COPD susceptibility genes;[34] and 5) we have data on the frequency of lower respiratory infections in the study subjects.

The major concern with unrecognized population stratification is that of inflation of the type 1 error rate. However, there is also a possibility of type 2 error and this may have influenced the lack of association observed in this study. The use of unlinked 'genomic control' markers may have allowed this issue to be examined further. Unfortunately, genotyping of the requisite number of neutral, genomic control loci to measure stratification was beyond the scope of this study.

Analysis of individual polymorphisms and haplotypes did not suggest any associations between the polymorphisms/haplotypes and an increased rate of decline in lung function in smokers. In addition, the polymorphisms were not important predictors of susceptibility to infection, suggesting that they do not influence DEFA1/DEFA3-mediated or DEFB1-mediated responses to infection.

Power analysis indicates that our study was not underpowered. We estimated that for the DEFA1/DEFA3 copy number polymorphism, the study design has eighty percent power to detect an association with a relative risk of 1.98. For the DEFB1 -20, -44, and -52 SNPs, the study design has eighty percent power to detect associations with relative risks of 1.60, 1.72, and 1.61, respectively.

We found seven DEFB1 haplotypes with estimated frequencies ranging from <1.0% to 42.3% in the subject group with no decline in lung function. These results are similar to previously reported data . Some of the haplotypes reported in our study are very rare. In the studies by Dork et al.[28] and Matsushita et al.[17] where there was a total of 103 German blood donors and 60 Japanese patients with COPD, respectively, they may not have had large enough sample sizes to detect rare haplotypes. In addition, racial/ethnic differences may account for the differences observed.

It is known that the frequencies of DEFB1 SNPs differ between racial/ethnic groups.[30] We determined the prevalence of the genotypes across white and African American subjects and found that the DEFB1 G-52A SNP differed significantly between white and African American populations. In addition, we did not detect the G allele of the DEFB1 C-44G SNP in twenty-seven African American subjects suggesting that this allele is very rare or non-existent in the African American population. It has been shown that the C-44G SNP is monomorphic in the African American population .

In interpreting our data, a few limitations should be considered. To our knowledge, this is the first time the DEFA1/DEFA3 copy number polymorphism and the three DEFB1 SNPs have been tested for association with rate of decline in lung function in any population. Although we did not find association between the DEFA1/DEFA3 copy number polymorphism or DEFB1 polymorphisms and rate of decline in lung function, these polymorphisms may influence other COPD-related phenotypes. Furthermore, as we have only examined a few polymorphisms, we have not ruled out the possibility that other genetic variation in the alpha- and beta-defensins genes may be associated with the disease. The genetic basis of COPD may differ between racial/ethnic groups. We limited the association study to white subjects and some of the genotype frequencies differ significantly between white and African American racial groups. Therefore, it may be of interest to investigate the role of these polymorphisms in other racial/ethnic populations.

## Conclusion

Variation in the DEFB1 gene is associated with COPD in both Japanese and Chinese populations. Our results indicate that in a white population, the DEFA1/DEFA3 copy number polymorphism and the three common DEFB1 SNPs at positions -20, -44, and -52 do not influence smokers' susceptibility to lung function decline or susceptibility to lower respiratory infection.

## Competing interests

The author(s) declare that they have no competing interests.

## Authors' contributions

AW carried out the molecular genetic studies and drafted the manuscript. JH and JR carried out some of the genotyping assays. KB performed the statistical analysis and helped revise the manuscript. JC, NA, PP, and AS contributed to the study conception and design, sample acquisition, analysis and interpretation of the data, and helped revise the manuscript. All authors read and approved the final manuscript.
